# Reference ranges for myocardial native T1, T2, and extracellular volume at 5.0T cardiac magnetic resonance imaging in healthy adults

**DOI:** 10.1016/j.jocmr.2026.102753

**Published:** 2026-06-04

**Authors:** Shiyu Wang, Xianling Qian, Yali Wu, Xiyin Miao, Ziyun Guan, Dong Wang, Rui Wang, Yinyin Chen, Ling Chen, Zhuolin Liu, Shaofeng Duan, Lin Tian, Hang Jin, Mengsu Zeng

**Affiliations:** a Department of Radiology, Zhongshan Hospital, Fudan University, Shanghai, China; b Shanghai Institute of Medical Imaging, Shanghai, China; c United Imaging Healthcare, Shanghai, China; d Collaborative Innovation Department, United Imaging Healthcare, Shanghai, China; e Circle Cardiovascular Imaging Inc., Calgary, Alberta, Canada

**Keywords:** CMR, 5.0T, T1 mapping, T2 mapping, extracellular volume

## Abstract

**Background:**

A 5.0T Cardiovascular magnetic resonance (CMR) offers potential advantages over 3.0T systems, including improved signal-to-noise ratio and spatial resolution. Although myocardial T1 and T2 mapping techniques are increasingly applied in clinical practice, reference ranges for these parameters at 5.0T have not yet been established. The aim of this study is to determine normal reference values for myocardial native T1, T2 relaxation times, and extracellular volume fraction (ECV) in healthy adults using 5.0T CMR.

**Methods:**

This prospective study enrolled 181 healthy Chinese adults aged 20–80 years who underwent 5.0T CMR. T1 and T2 mapping of the left ventricle were performed using a motion-corrected modified Look-Locker inversion recovery sequence and a T2-prepared single-shot sequence, respectively. Global and segmental myocardial T1, T2, and ECV values were quantified. Associations with sex, age, body mass index (BMI), and myocardial thickness were analyzed.

**Results:**

The mean global myocardial T1, T2, and ECV values were 1496 ± 37 ms, 35 ± 3 ms and 27 ± 2 %, respectively. Females showed significantly higher T1 and T2 values than males (T1: 1509 ± 35 ms vs 1483 ± 35 ms, *p*<.001; T2: 36 ± 2 ms vs 34 ± 2 ms, *p*<.001). No significant age-related differences were observed. T1, T2, and ECV values were inversely associated with BMI (T1: *r*=-0.16, *p*=.034; T2: *r*=-0.35, *p*<.001; ECV: *r*=-0.31, *p*=.015). T1 values positively correlated with myocardial thickness (*r*=0.19, *p*<.001), whereas T2 and ECV values negatively correlated with thickness (*r*=-0.21 and −0.19, respectively; both *p*<.001). Segmental analysis showed lower T1 but higher T2 and ECV values at the apex compared with the mid and basal myocardium (all *p*<.001).

**Conclusion:**

This study establishes normative myocardial T1, T2, and ECV values at 5.0T CMR in healthy adults. Myocardial tissue characteristics were influenced by sex, BMI, and myocardial thickness, with regional variations observed across myocardial segments.

## Graphical abstract

**Figure:**
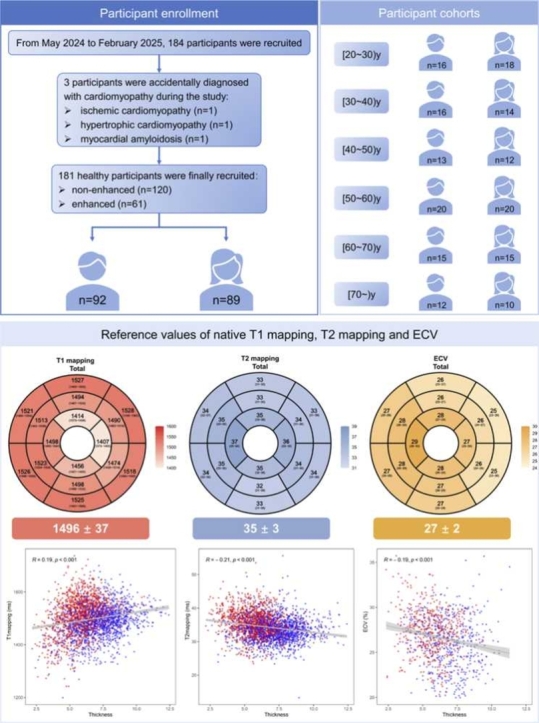

## 1 Introduction

Cardiovascular magnetic resonance (CMR) is established as the reference standard for noninvasive myocardial tissue characterization [1], with increasing integration into clinical practice. Quantitative mapping techniques enable pixel-wise assessment of T1 and T2 relaxation times [2], addressing the limitations of late gadolinium enhancement (LGE) and conventional sequences that rely on subjective visual evaluation of signal intensity differences [3]. T1 and T2 mapping have been validated as reliable surrogate markers for detecting diffuse fibrosis, edema, and lipid accumulation [4], [5], [6], while T1-derived extracellular volume fraction (ECV) fraction permits quantification of both focal and diffuse myocardial fibrosis [7], [8].

The recent development of 5.0T MR systems heralds a new era in CMR. Studies by Lin *et al*. have reported that cine imaging at 5.0T provides superior image quality and more accurate functional quantification compared with 3.0T imaging [9]. Our previous studies further demonstrated that 5.0T CMR offers superior image quality for both LGE imaging and coronary MR angiography compared with 3.0T MR [10], [11]. The integration of T1 and T2 mapping techniques at 5.0T addresses a critical gap in the clinical application of ultrahigh-field CMR, highlighting the urgent need to define field strength-specific normative ranges for native T1 and T2 values to enhance pathological detection and standardization, which is influenced by multiple factors, including magnetic field strength, MRI vendor differences, and population demographics. While normal values for T1 and T2 relaxation times have been well established at 1.5T and 3.0T, corresponding data at 5.0T remain unavailable. Therefore, there is an urgent need to define field strength-specific normative ranges for parametric mapping at 5.0T to facilitate and standardize its clinical application.

The purpose of this study was to establish reference ranges for myocardial T1 and T2 relaxation times and ECV at 5.0T in healthy subjects across different age groups, and to identify factors independently associated with these parameters.

## 2 Materials and methods

### 2.1 In vitro phantom experiments

#### 2.1.1 Imaging protocol

In vitro imaging was performed using the National Institute of Standards and Technology (NIST)/ International Society for Magnetic Resonance in Medicine (ISMRM) system phantom on a 5.0T MR scanner (uMR Jupiter; United Imaging, Shanghai, China). The imaging protocol included Inversion Recovery Fast Spin-Echo T1-weighted imaging (IR-FSE T1), Modified Look-Locker Inversion Recovery (MOLLI) T1 mapping with a 5(3)3 acquisition scheme, Spin-Echo Multi-Echo T2-weighted imaging (SEME T2), and T2-prepared single-shot imaging. Detailed imaging parameters for each sequence are provided in Table S1.

#### 2.1.2 Image analysis

T1 relaxation times of the NIST/ISMRM phantom spheres were measured using both the IR-FSE T1 sequence and the MOLLI T1 mapping sequence. T2 relaxation times were measured using the SEME T2 sequence and T2-prepared single-shot imaging. T1 and T2 values were recorded separately for each sphere of the NIST/ISMRM phantom.

### 2.2 In vivo experiments

#### 2.2.1 Study participants

This prospective study was approved by the institutional ethics committee (approval no. B2023–371) and conducted in accordance with the Declaration of Helsinki. Written informed consent was obtained from all participants prior to CMR examinations. Between May 2024 and February 2025, healthy asymptomatic participants aged 20–80 years were recruited through community advertisements. Participants were stratified by age group at 10-year intervals, with an approximately 1:1 ratio of males to females within each group. Individuals without a history of cardiovascular disease, conditions affecting the cardiovascular system, or contraindications to MRI were prospectively enrolled. General clinical information, including age, sex, height, weight, and personal and family history, was collected.

Eligible participants had no history of cardiovascular disease, no clinically documented systemic disease affecting the cardiovascular system, and no contraindications to CMR. Hypertension was defined as a prior clinical diagnosis, current use of antihypertensive medication, or systolic and/or diastolic blood pressure ≥140/90 mmHg based on home blood pressure monitoring. Diabetes mellitus and hypercholesterolemia were excluded based on documented clinical diagnoses, use of glucose- or lipid-lowering medications, or abnormal laboratory findings from routine health examinations within the preceding 6 months. Additional exclusion criteria included a family history of cardiovascular disease (e.g., acute coronary syndrome, coronary atherosclerotic heart disease, or congenital heart disease), active smoking, professional athletic participation or long-term endurance training, history of malignancy, clinically confirmed hyperthyroidism or hypothyroidism, and severe hepatic or renal dysfunction. All participants underwent transthoracic echocardiography to exclude subclinical cardiomyopathy prior to CMR.

Participants willing to undergo contrast-enhanced examination received cardiac contrast-enhanced CMR examinations, while the others underwent non-contrast examinations. Those who underwent contrast-enhanced CMR additionally underwent blood sampling for hematocrit measurement.

#### 2.2.2 CMR acquisition

All CMR examinations were performed using a 5.0T MR scanner (uMR Jupiter; United Imaging, Shanghai, China) equipped with a 24-channel body coil. Standard cine imaging was acquired to assess left ventricular and right ventricular myocardial function. For each participant, 7–10 consecutive short-axis slices (8 mm thickness) covering the ventricles and long-axis 2-, 3-, and 4-chamber images were obtained. T1 mapping was performed using a MOLLI sequence with a 5(3)3 acquisition scheme. T1 mapping was acquired at five short-axis levels: the base, between the base and mid-ventricle, mid-ventricle, between the mid-ventricle and apex, and apex. T2 mapping was performed using a T2-prepared single-shot sequence at the same short-axis locations as T1 mapping.

In participants undergoing contrast-enhanced imaging, gadodiamide (Omniscan; GE HealthCare, Chalfont-St. Giles, United Kingdom) was administered at a dose of 0.15 mmol/kg body weight, injected at a rate of 2.0 mL/sec. Eight minutes after contrast injection, two-dimensional short- and long-axis LGE images were acquired using a phase-sensitive inversion recovery (PSIR) sequence. Post-contrast T1 mapping was then performed with the same imaging prescription as pre-contrast T1 mapping, approximately 12 min after gadolinium administration. Imaging parameters for T1 and T2 mapping are detailed in Table S2.

#### 2.2.3 CMR analysis

All images were analyzed using the software CVI42 (Circle Cardiovascular Imaging, Calgary, Alberta, Canada). Image analysis and the qualitative evaluation of overall image quality were independently performed by two investigators (X.L.Q. and S.Y.W., with 5 and 10 years of CMR experience, respectively). Intra-observer variability was assessed by re-analysis of image quality by a single investigator after a one-month interval.

End-diastolic volume (EDV), end-systolic volume (ESV), and ejection fraction (EF), and cardiac output (CO) for the left and right ventricle were quantified from cine images. Ventricular volumes were indexed to the body surface area (BSA) using the Mosteller formula. Left ventricular wall thickness at end-diastole was measured for each segment according to the American Heart Association (AHA) 16-segment model.

T1 and T2 values of the left ventricle were measured according to the Society for Cardiovascular Magnetic Resonance (SCMR) post-processing guidelines [12]. To minimize partial volume effects from the blood pool, 10% of the subendocardial and subepicardial borders were manually excluded. T1 and T2 values were additionally assessed on a per-segment basis. In a subset of 61 subjects, ECV fraction was measured. The partition coefficient lambda (λ) and ECV were calculated as follows:

$$\lambda =\frac{\;1/T1\mathrm{myocardium}\;\mathrm{postC}-1/T1\mathrm{myocardium}\;\mathrm{perC}}{1/T1\mathrm{blood}\;\mathrm{postC}-1/T1\mathrm{blood}\;\mathrm{perC}}$$

$$\mathrm{ECV}=\left(1-\mathrm{Hematocrit}\right)\times \lambda$$

Overall image quality was subjectively evaluated for both global and segmental myocardium using a five-point Likert scale (1 = poor, nondiagnostic; 2 = fair, noticeable motion artifacts or distortion but partially diagnostic; 3 = adequate, moderate motion artifacts or distortion but sufficiently diagnostic; 4 = good, mild motion artifacts or distortion; 5 = excellent, minimal to no motion artifacts or distortion) [13]. The evaluation included myocardial structure delineation, motion artifacts, susceptibility effects, and image homogeneity. Each criterion was scored independently, and the lowest score among these parameters was assigned as the overall image quality score. As part of the standard quality control, goodness-of-fit (R^2^) analysis was performed for all T1 mapping images to assess the reliability of pixel-wise parameter estimation.

#### 2.2.4 Statistical analysis

All statistical analyses were performed using the R statistical computing environment (version 4.3.1; R Foundation for Statistical Computing, Vienna, Austria) and GraphPad Prism (version 8.0.2; GraphPad Software, San Diego, California). Continuous variables were expressed as mean ± standard deviation (SD) for normally distributed data or as median and interquartile range (IQR) for non-normally distributed data. Categorical variables were presented as frequencies with corresponding percentages.

Differences in T1 and T2 values obtained from the NIST/ISMRM phantom were analyzed using the paired *t* test. Study participants were stratified into decade-based age groups. Categorical variables across age groups were compared using the χ2 test or Fisher exact test, as appropriate. Continuous variables among different age groups were compared using analysis of variance (ANOVA) for normally distributed data or the Kruskal-Wallis test and Friedman test for non-normally distributed data. Comparisons between sex groups were performed using the Student *t* test for normally distributed variables or the Mann-Whitney *U* test for non-normally distributed variables. Interobserver agreement for measurements of left and right ventricular function, left ventricular wall thickness, T1 and T2 times, and ECV was evaluated using intraclass correlation coefficients (ICCs) and Bland-Altman analysis. Intra-observer agreement for measurements of T1 and T2 times, and ECV was evaluated using ICCs. For subsequent analyses, the average values from the two investigators were used. Bivariate correlations between myocardial parameters (T1 and T2 relaxation times, ECV) and continuous covariates (age, BSA, myocardial thickness, heart rate) were assessed using Pearson correlation coefficients. For comparisons of myocardial native T1, T2, and ECV values between the present 5.0T cohort and previously reported 1.5T and 3.0T values, independent samples *t* tests were used. Effect sizes were assessed by calculating Cohen’s d. All statistical tests were two-tailed, and a *p* value <.05 was considered to indicate statistical significance.

## 3 Results

### 3.1 Phantom study

Reference T1 values measured by IR-FSE T1 and MOLLI T1 mapping, and reference T2 values provided by SEME T2 and T2-prepared single-shot imaging for T2 mapping at 5.0T MR are summarized in Table S3, Table S4, and Figure S1. No significant differences in T1 and T2 values were observed between the different sequences (*p* =.998 anxd *p* =.997, respectively).

### 3.2 Characteristics of study participants

**Fig. 1:**
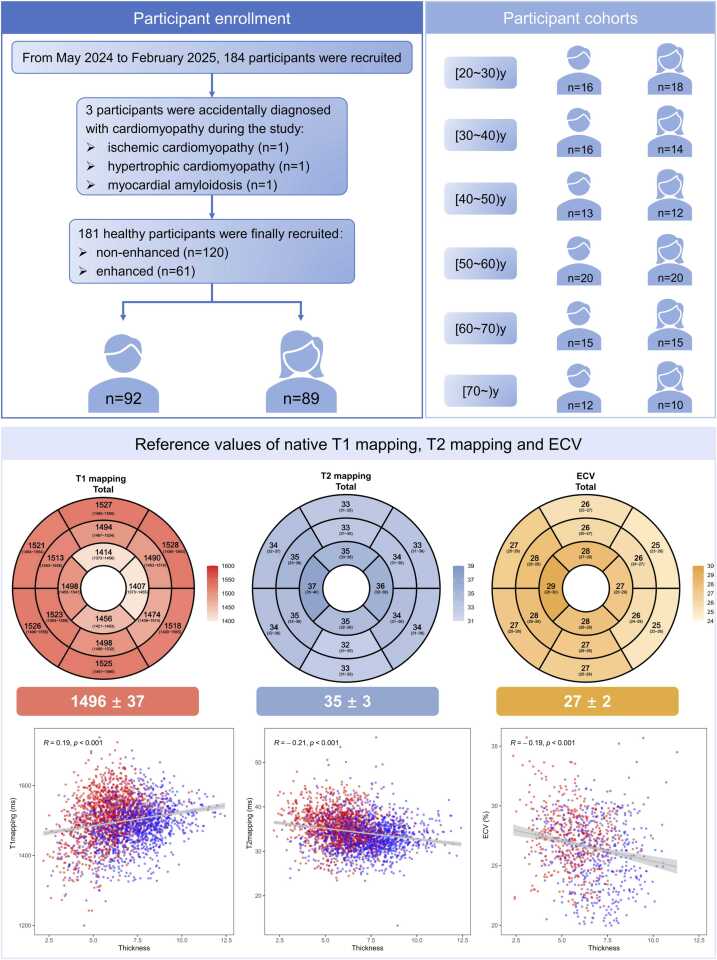
Flowchart of participant enrollment, cohort distribution, and main study findings. A total of 184 participants were recruited between May 2024 and February 2025. Three participants were excluded due to incidental diagnoses of cardiomyopathy, resulting in 181 healthy participants. Participants were divided into non-contrast (*n* = 120) and contrast-enhanced (*n* = 61) CMR groups. The distribution of participants by age group and sex is shown. Reference values for native T1, T2, and ECV were established, and their correlations with myocardial thickness were analyzed. *ECV* extracellular volume fraction, *CMR* cardiovascular magnetic resonance

A total of 184 subjects were screened, among whom three were excluded due to incidental findings of ischemic cardiomyopathy (ICM), hypertrophic cardiomyopathy (HCM), or myocardial amyloidosis. Ultimately, 181 participants were enrolled in the study, comprising 51% (92/181) males and 49% (89/181) females. Among them, 60 volunteers underwent contrast-enhanced examination. The distribution of male and female participants across different age groups is shown in Fig. 1. Baseline demographic and CMR characteristics are summarized in Table 1. All participants were of Chinese ethnicity. In all participants with normal cardiac function, enhanced CMR showed no focal myocardial scarring on LGE sequences.

**Table 1:** Baseline characteristics of the participant population by age group

Characteristics	Total population (*n* = 181)	[20 ∼ 30) (*n* = 34)	[30 ∼ 40) (*n* = 30)	[40 ∼ 50) (*n* = 25)	[50 ∼ 60) (*n* = 40)	[60 ∼ 70) (*n* = 30)	[70 ∼) (*n* = 22)	*p* value
Age (years)^a^	51 (33 ∼ 60)	24 (23 ∼ 26)	33.5 (32 ∼ 35)	44 (41 ∼ 48)	55 (54 ∼ 57)	63.5 (60.25 ∼ 66)	72 (72 ∼ 74)	<0.001
Gender^b^								0.995
Male	92 (50.83)	16 (47.06)	16 (53.33)	13 (52.00)	20 (50.00)	15 (50.00)	12 (54.55)	
Female	89 (49.17)	18 (52.94)	14 (46.67)	12 (48.00)	20 (50.00)	15 (50.00)	10 (45.45)	
Weight (kg)^c^	65.83±12.50	62.49±11.60	70.70±14.64	70.32±12.59	66.39±11.75	62.63±9.33	62.57±13.12	0.01
Height (cm)^c^	166.39±8.57	168.85±6.43	169.40±7.89	167.84±8.47	164.70±8.95	163.73±8.78	163.50±9.61	0.01
BSA (m^2^)^c^	1.74±0.20	1.71±0.17	1.82±0.23	1.81±0.20	1.74±0.19	1.68±0.16	1.68±0.22	0.02
BMI (kg/m^2^)^c^	23.63±3.13	21.85±3.42	24.40±3.38	24.83±3.30	24.33±2.81	23.26±1.92	23.19±2.88	<0.001
HR (bpm)^c^	65.82±9.99	66.21±11.96	68.03±8.92	67.56±9.03	64.67±9.48	64.77±9.28	63.77±11.11	0.54

Note: *BSA* body surface area, *BMI* body mass index, *HR* heart rate

a Data are medians with interquartile ranges in parentheses

b Data are the number of participants with the percentage in parentheses

c Data are means ± standard deviations

**Table 2:** Intraclass correlation coefficients for cardiac function, native T1 mapping, T2 mapping, and extracellular volume measurements

Measurements	Reader 1	Reader 2	ICC
*Left ventricle*			
EDVi (mL/m^2^)^a^	62.34 (56.00 ∼ 69.80)	63.13 (56.07 ∼ 70.46)	0.99
ESVi (mL/m^2^)^a^	23.23 (19.74 ∼ 26.62)	23.39 (20.02 ∼ 27.20)	0.99
SVi (mL/m^2^)^a^	38.90 (34.66 ∼ 43.83)	39.27 (34.94 ∼ 44.14)	0.98
EF (%)^b^	62.56±5.42	62.44±5.39	0.97
CI (L/min/m^2^)^a^	2.48 (2.15 ∼ 2.86)	2.52 (2.19 ∼ 2.90)	0.99
*Right ventricle*			
EDVi (mL/m^2^)^b^	70.60±13.36	70.29±13.58	0.97
ESVi (mL/m^2^)^b^	32.25±8.30	32.07±8.17	0.99
SVi (mL/m^2^)^b^	38.34±7.48	38.42±7.54	0.97
EF (%)^b^	54.64±6.10	54.79±6.02	0.97
CI (L/min/m^2^)^a^	2.39 (2.06 ∼ 2.82)	2.41 (2.06 ∼ 2.86)	0.97
*Native T1 mapping*			
mean values (msec)^b^	1494.45±37.72	1497.20±36.40	0.98
SD values (msec)^b^	108.54±25.50	103.54±23.78	0.94
*T2 mapping*			
mean values (msec)^b^	34.62±2.68	34.43±2.57	0.98
SD values (msec)^a^	6.60 (5.84 ∼ 7.28)	6.28 (5.65 ∼ 6.99)	0.29
*ECV*			
mean values (%)^b^	26.70±2.03	26.49±2.02	0.98
SD values (%)^b^	3.07±1.12	2.98±1.09	0.98

Note: *ICC* intraclass correlation coefficient, *EDVi* end-diastolic volume index, *ESVi* end-systolic volume index, *SVi* stroke volume index, *EF* ejection fraction, *CI* cardiac index, *ECV* extracellular volume fraction, *SD* standard deviation

a Data are medians with interquartile ranges in parentheses

b Data are means ± standard deviations

### 3.3 Reproducibility and overall image quality of myocardial T1, T2, and ECV

Inter- and intra-observer reproducibility for myocardial tissue parameter assessments is summarized in Table S5. Bland-Altman plots for myocardial thickness (Figure S2), native T1 (Figure S3), T2 (Figure S4), and ECV (Figure S5) values, assessed according to the AHA 16-segment model, are also provided. The intraclass correlation coefficients (ICCs) for global T1, T2 values, and ECV were all 0.98. The ICC values for each segment are summarized in Table S6. Overall, segmental and global myocardial T1, T2, and ECV mappings demonstrated good consistency for all measures. However, segment-dependent variability was observed, particularly for T1 and T2 mapping, even though some of these differences did not reach statistical significance. Among all segments, the septal regions demonstrated the highest and most consistent reproducibility, whereas the inferior wall showed comparatively lower reproducibility (T1: 0.99 [0.96, 0.99] vs 0.95 [0.92, 0.96], *p* = .27; T2: 0.98 [0.97, 0.99] vs 0.95 [0.91, 0.96], *p* = .20). In addition, midventricular segments exhibited the most robust and stable measurements, while apical segments showed the lowest reproducibility (T1: 0.98 ± 0.02 vs 0.91 ± 0.04, *p* = .001; T2: 0.98 ± 0.02 vs 0.94 ± 0.03, *p* = .020).

The overall image quality scores of segmental and global myocardial T1, T2, and ECV mappings are detailed in Table S7 and Figure S6, with a representative scoring example provided in Figure S6. Regarding the quantitative quality control, the analysis revealed an exceptionally high goodness-of-fit for T1 mapping across all evaluated subjects. Specifically, the R^2^ values for all T1 mapping images were strictly > 0.95, indicating highly reliable pixel-wise parameter estimation at 5T. The detailed segmental R^2^ values for T1 mapping are summarized in Table S8.

### 3.4 Normal myocardial T1, T2, and ECV values and their relation to age, sex, heart rate, and BMI

Excellent intra- and interobserver reproducibility was demonstrated for the quantitative assessment.

**Fig. 2:**
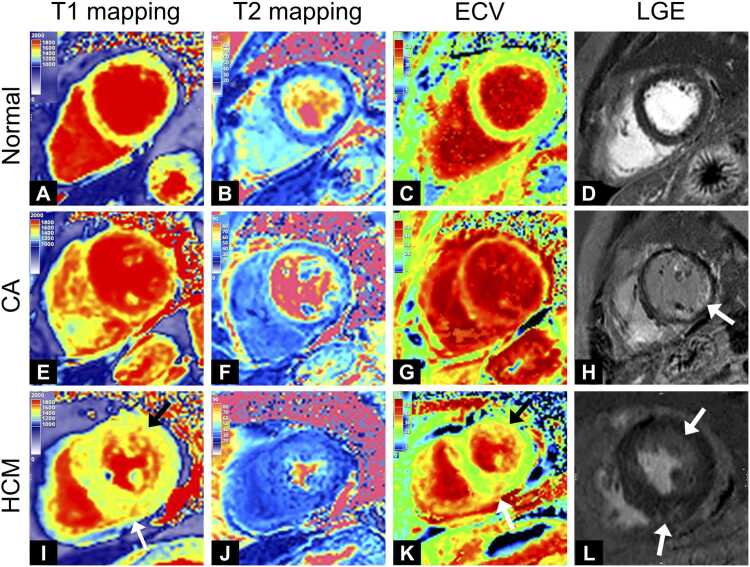
Representative native T1 mapping, T2 mapping, ECV mapping, and LGE images in a healthy participant, a patient with CA, and a patient with HCM. (A–D) Images from a 49-year-old healthy woman. The global myocardial T1 value (A), T2 value (B), and ECV (C) were 1509 ± 132 ms, 32 ± 4 ms, and 26 ± 2%, respectively; no abnormal enhancement was observed on LGE imaging (D). (E–H) Images from a 74-year-old woman with CA. Myocardial T1 values were diffusely elevated [ E, 1656 ± 89 ms (the normal reference T1 value is 1496 ± 37 ms), which was ∼10% higher than the 95th percentile in healthy controls], ECV was markedly increased [G, 40 ± 6% (the normal reference ECV is 27 ± 2%), which was ∼40% higher than the 95th percentile in healthy controls], and diffuse subendocardial fibrosis was evident on LGE imaging (H, white arrow). (I–L) Images from a 67-year-old man with HCM. Myocardial T1 values were elevated at the anterolateral wall (I, 1592 ± 62 ms, which was ∼6% higher than the 95th percentile in healthy controls; K, ECV 30 ± 3%, which was ∼7% higher than the 95th percentile in healthy controls; black arrow) and at the inferior right ventricular insertion point (I, 1600 ± 59 ms, which was ∼7% higher than the 95th percentile in healthy controls; K, ECV 30 ± 3%, which was ∼7% higher than the 95th percentile in healthy controls; white arrow), with corresponding delayed enhancement on LGE imaging (L, white arrow). The native T1 values demonstrated good agreement with ECV values. *ECV* extracellular volume fraction, *LGE* late gadolinium enhancement, *CA* cardiac amyloidosis, *HCM* hypertrophic cardiomyopathy

Cardiac function measurements and normal myocardial native T1, T2, and ECV values for the 181 participants, stratified by decade-based age groups, are presented in Table 3. Across the total cohort, the mean native T1 value was 1496 ± 37 ms, the mean T2 value was 35 ± 3 ms, and the mean ECV was 27 ± 2%. Both native T1 and T2 values were significantly higher in female compared with male participants (1509 ± 35 ms vs 1483 ± 35 ms for T1; 36 ± 2 ms vs 34 ± 2 ms for T2; both *p* <.001). Representative cases of a healthy participant and cases of myocardial amyloidosis and HCM (Clinical diagnosis established in accordance with the 2023 European Society of Cardiology (ESC) Guidelines for the management of cardiomyopathy[14]) are shown in Fig. 2.

**Table 3:** Cardiac function measurements and normal myocardial native T1, T2, and extracellular volume values by age group

Values	Total population	[20 ∼ 30)	[30 ∼ 40)	[40 ∼ 50)	[50 ∼ 60)	[60 ∼ 70)	[70 ∼)
*Left ventricle*							
EDVi (mL/m^2^)^a^	62.81 (56.34 ∼ 70.10)	69.68 (60.07 ∼ 73.56)	66.00 (62.45 ∼ 74.61)	61.74 (53.23 ∼ 70.12)	62.61 (56.87 ∼ 68.39)	58.00 (51.76 ∼ 62.25)	60.20 (51.24 ∼ 63.84)
ESVi (mL/m^2^)^a^	23.30 (19.87 ∼ 26.90)	24.87 (22.03 ∼ 26.02)	23.40 (21.17 ∼ 29.07)	24.23 (20.56 ∼ 28.68)	23.66 (20.89 ∼ 27.04)	20.23 (19.04 ∼ 22.50)	19.50 (17.95 ∼ 23.65)
SVi (mL/m^2^)^a^	38.91 (34.82 ∼ 44.02)	42.82 (37.59 ∼ 46.69)	42.37 (38.94 ∼ 44.79)	37.53 (32.99 ∼ 42.59)	38.59 (34.32 ∼ 43.62)	37.02 (32.42 ∼ 41.42)	37.06 (35.02 ∼ 43.18)
EF (%)^b^	62.50±5.37	62.30±5.30	62.94±4.61	60.86±5.40	61.87±5.01	63.18±5.26	64.35±6.90
CI (L/min/m^2^)^a^	2.50 (2.17 ∼ 2.89)	2.66 (2.29 ∼ 2.95)	2.72 (2.50 ∼ 3.16)	2.64 (2.21 ∼ 2.89)	2.39 (2.13 ∼ 2.76)	2.31 (1.97 ∼ 2.47)	2.22 (2.09 ∼ 2.66)
*Right ventricle*							
EDVi (mL/m^2^)^b^	70.44±13.36	76.25±13.13	74.86±11.55	71.08±12.24	70.68±14.38	61.45±11.74	66.38±10.51
ESVi (mL/m^2^)^b^	32.16±8.21	35.58±8.09	34.87±6.83	33.56±7.23	32.40±8.85	26.66±7.01	28.51±6.99
SVi (mL/m^2^)^b^	38.38±7.46	40.67±8.25	39.97±6.93	38.26±7.73	38.27±7.71	34.79±6.44	37.86±6.01
EF (%)^b^	54.71±6.02	53.39±6.37	53.47±4.90	53.35±6.61	54.55±5.92	56.95±5.49	57.35±5.93
CI (L/min/m^2^)^a^	2.40 (2.05 ∼ 2.82)	2.64 (2.23 ∼ 2.86)	2.57 (2.31 ∼ 3.01)	2.59 (2.24 ∼ 2.88)	2.28 (2.11 ∼ 2.71)	2.08 (1.89 ∼ 2.44)	2.35 (2.02 ∼ 2.70)
*Native T1 mapping*							
mean values (msec)^b^	1495.83±36.91	1494.27±43.88	1493.32±34.34	1512.06±39.60	1499.18±29.13	1481.16±38.15	1497.99±31.34
SD values (msec)^b^	106.04±24.29	117.04±27.27	112.55±25.10	104.06±27.21	100.88±21.18	99.46±20.20	100.46±19.28
*T2 mapping*							
mean values (msec)^b^	34.52±2.61	34.90±2.87	34.94±2.72	33.75±2.77	34.62±2.36	34.62±2.13	33.92±2.90
SD values (msec)^a^	6.51 (5.79 ∼ 7.19)	6.77 (6.30 ∼ 7.28)	6.35 (5.81 ∼ 7.42)	6.57 (5.84 ∼ 7.23)	6.32 (5.82 ∼ 7.10)	6.10 (5.48 ∼ 6.75)	6.99 (5.77 ∼ 7.20)
*ECV*							
mean values (%)^b^	26.60±2.01	26.31±1.57	26.94±1.87	27.39±1.39	26.24±2.76	26.89±1.90	26.10±2.36
SD values (%)^b^	3.03±1.10	3.26±0.63	3.31±1.36	3.20±1.16	2.70±1.44	2.84±0.97	2.76±0.83

Note: *EDVi* end-diastolic volume index, *ESVi* end-systolic volume index, *SVi* stroke volume index, *EF* ejection fraction, *CI* cardiac index, *ECV* extracellular volume fraction, *SD* standard deviation

a Data are medians with interquartile ranges in parentheses

b Data are means ± standard deviations

**Fig. 3:**
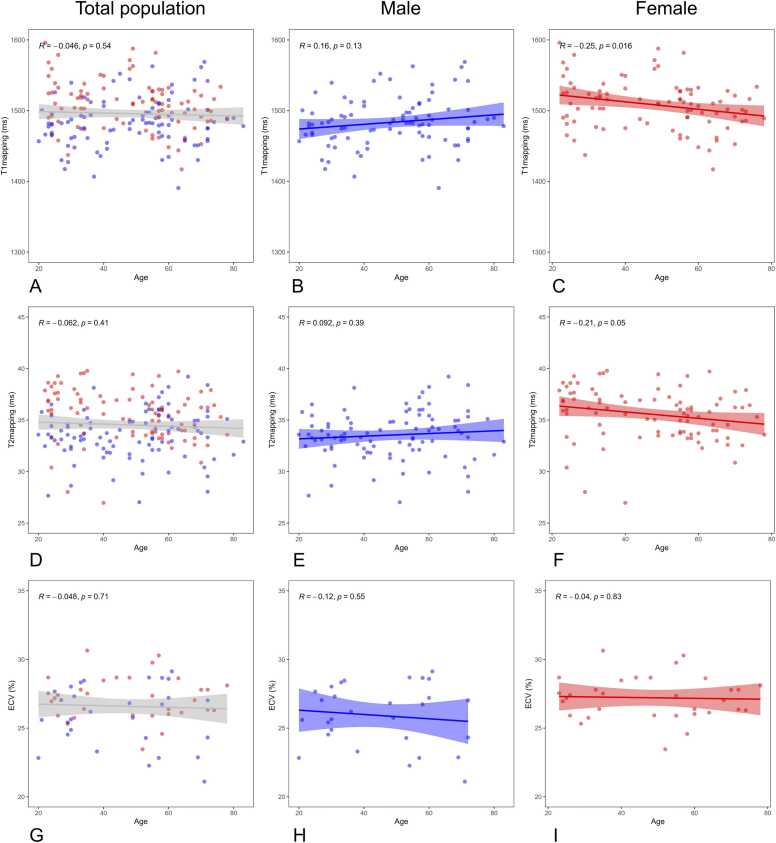
Regression plots of myocardial native T1 values, T2 values, and ECV according to age. Scatterplots show the relationship between age and native T1 values (A-C), T2 values (D-F), and ECV values (G-I) in the total cohort (A, D, G), male participants (B, E, H), and female participants (C, F, I), respectively. *ECV* extracellular volume fraction

**Fig. 4:**
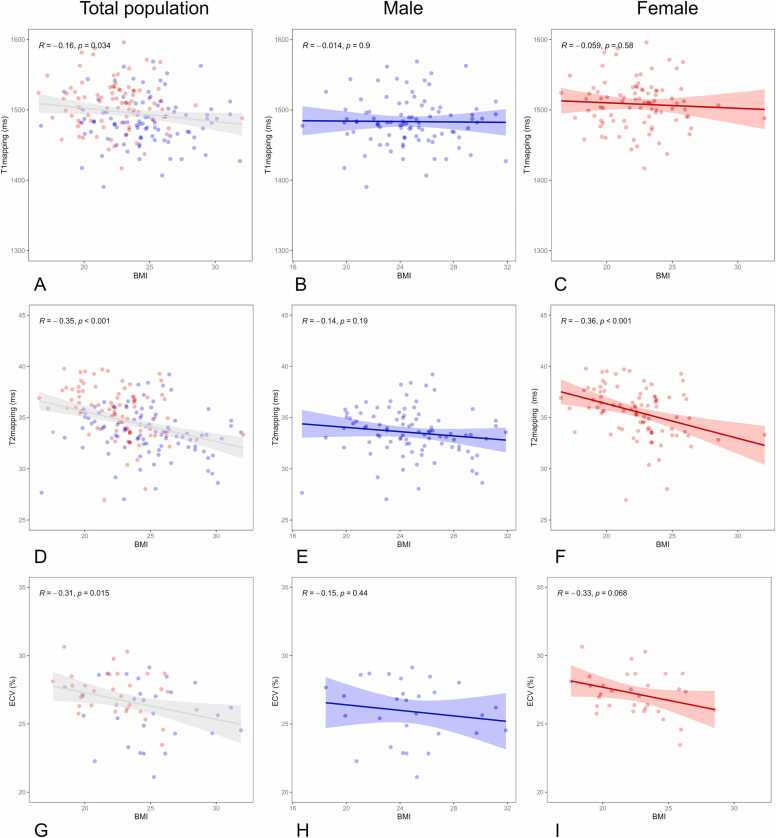
Regression plots of myocardial native T1 values, T2 values, and ECV according to BMI. Scatterplots show the relationship between BMI and native T1 values (A-C), T2 values (D-F), and ECV values (G-I) in the total cohort (A, D, G), male participants (B, E, H), and female participants (C, F, I), respectively. *ECV* extracellular volume fraction, *BMI* body mass index

**Fig. 5:**
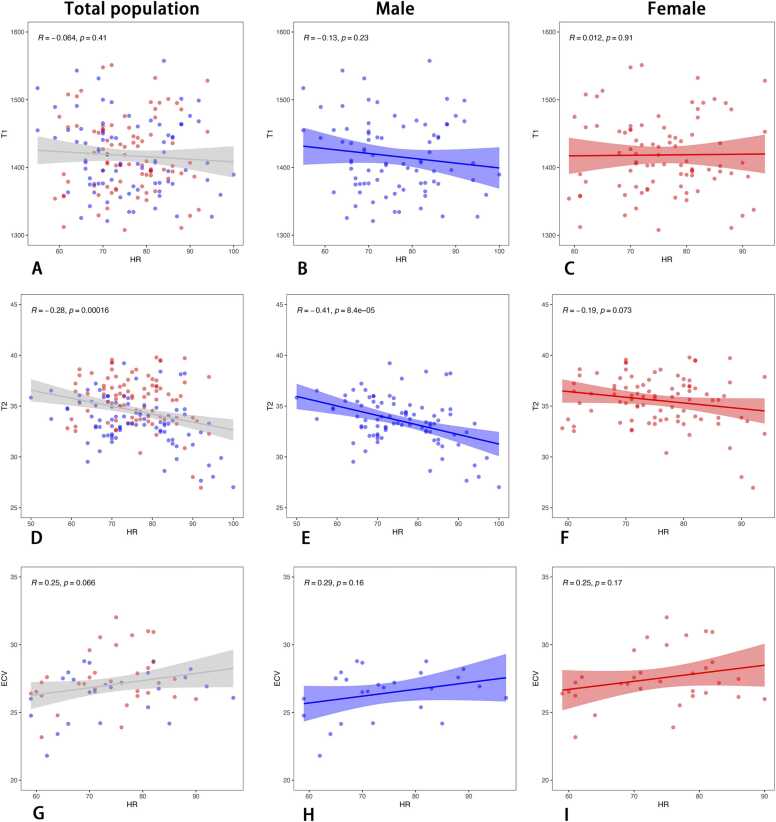
Regression plots of myocardial native T1 values, T2 values, and ECV according to HR. Scatterplots show the relationship between HR and native T1 values (A-C), T2 values (D-F), and ECV values (G-I) in the total cohort (A, D, G), male participants (B, E, H), and female participants (C, F, I), respectively. *HR* Heart rate, *ECV* extracellular volume fraction

The relationships between myocardial native T1, T2, and ECV values and age (Fig. 3), BMI (Fig. 4) and heart rate (Fig. 5) are also shown. Across all participants, linear regression analysis demonstrated no significant correlation between native T1, T2, or ECV values and age. However, in female participants, a negative correlation was observed between native T1 value and age (*r* = −0.25, *p* =.016). Native T1, T2, and ECV values were significantly negatively correlated with BMI across the total cohort (*r* = −0.16, *p* =.034 for T1; *r* = −0.35, *p* <.001 for T2; *r* = −0.31, *p* =.015 for ECV). A significant negative correlation between T2 value and BMI was observed in female participants (*r* = −0.36, *p* <.001), but not in male participants. Correlations between BMI and T1 or ECV values were not significant when analyzed separately by sex. Across all participants, linear regression analysis revealed no significant correlation between heart rate and either native T1 or ECV values. In contrast, a significant negative correlation was identified between heart rate and T2 values (*r = -*0.28, *p* <.001).

An exploratory analysis assessing the feasibility of estimating synthetic extracellular volume (ECV) from blood pool T1, including its correlations and agreement is detailed in the Supplementary Material (Supplementary Text S1).

### 3.5 Segmental analysis of myocardial T1, T2, and ECV values

**Fig. 6:**
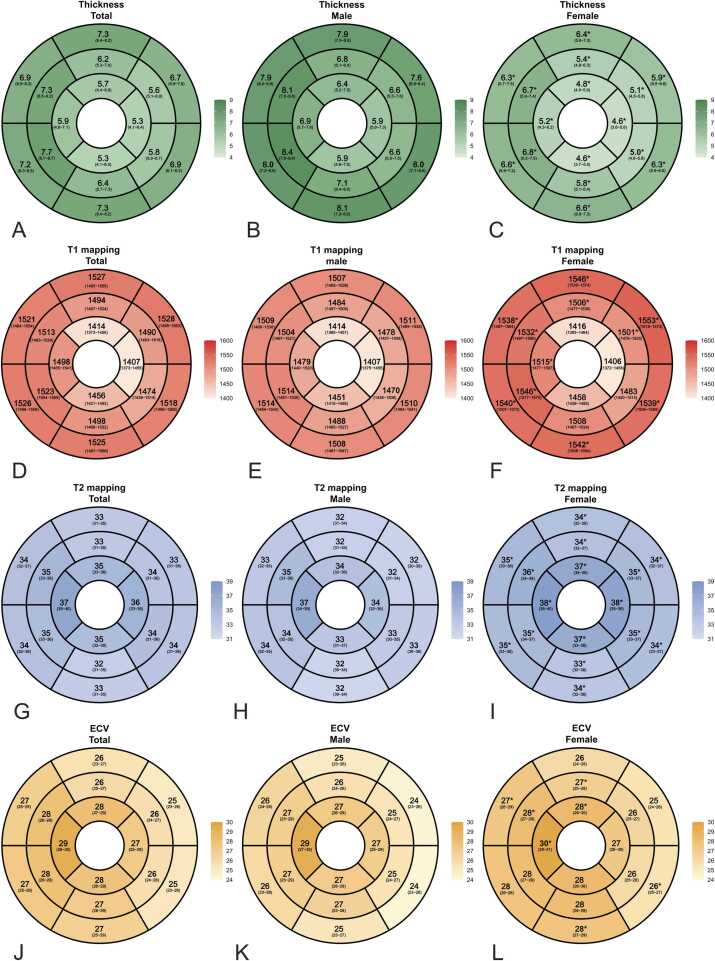
Bullseye plots illustrating myocardial thickness, native T1, T2, and extracellular volume fraction (ECV) values across the American Heart Association (AHA) 16-segment model. Segmental myocardial thickness (A-C), native T1 values (D-F), T2 values (G-I), and ECV values (J-L) are displayed for the total cohort, male participants, and female participants, respectively. *p <.05 for comparison between male and female participants

A total of 2.1% (62/2896) myocardial segments for T1 mapping and 1.8% (52/2896) myocardial segments for T2 mapping were excluded. No segment was excluded from post-contrast T1 mapping and ECV maps. Excluded segments were predominantly located in the inferior and apical regions and were mainly affected by motion artifacts, susceptibility-related signal loss, or severe image inhomogeneity. Segmental myocardial T1, T2, and ECV values, as well as myocardial thickness, according to the AHA 16-segment model are presented as bullseye plots in Fig. 6. Comprehensive pairwise comparisons across the 16 AHA segments revealed statistically significant intersegmental differences in T1, T2, and ECV values, objectively confirming the presence of regional myocardial heterogeneity (Figure S7). Segments were categorized into basal, midventricular, and apical regions following the AHA 16-segment model. Sectional variations in T1, T2, and ECV values from base to apex were evaluated and are summarized in Table 4. T2 and ECV values were higher at the apex compared with the mid and basal regions, whereas T1 values were lower at the apex.

**Table 4:** T1, T2, and extracellular volume values of participants according to left ventricular sections

Parameters	Base	Middle	Apex	*p* value
T1 mapping (msec)				
Total (*n*=181)	1527.54±41.38	1500.58±38.46	1446.57±49.21	<.001
Male (*n*=89)	1510.72 (1489.75 ∼ 1527.37)	1488.63±38.01	1437.63±44.24	<.001
Female (*n*=92)	1541.64±39.85	1512.08±35.44	1455.20±52.38	<.001
T2 mapping (msec)				
Total (*n*=181)	33.84±2.96	34.05±2.60	35.85±3.36	<.001
Male (*n*=89)	32.82±2.45	33.12±2.29	34.61±3.24	<.001
Female (*n*=92)	34.83±3.08	34.96±2.58	37.05±3.02	<.001
ECV (%)				
Total (*n*=61)	25.90±2.16	26.52±2.05	27.95 (26.95 ∼ 29.18)	<.001
Male (*n*=29)	25.18±2.46	25.83±2.38	27.10±2.16	<.001
Female (*n*=32)	26.53±1.67	27.15±1.46	28.56±2.12	.01

Data are presented as means ± standard deviations, or medians with interquartile ranges in parentheses, as appropriate

Note: *ECV* extracellular volume fraction

**Fig. 7:**
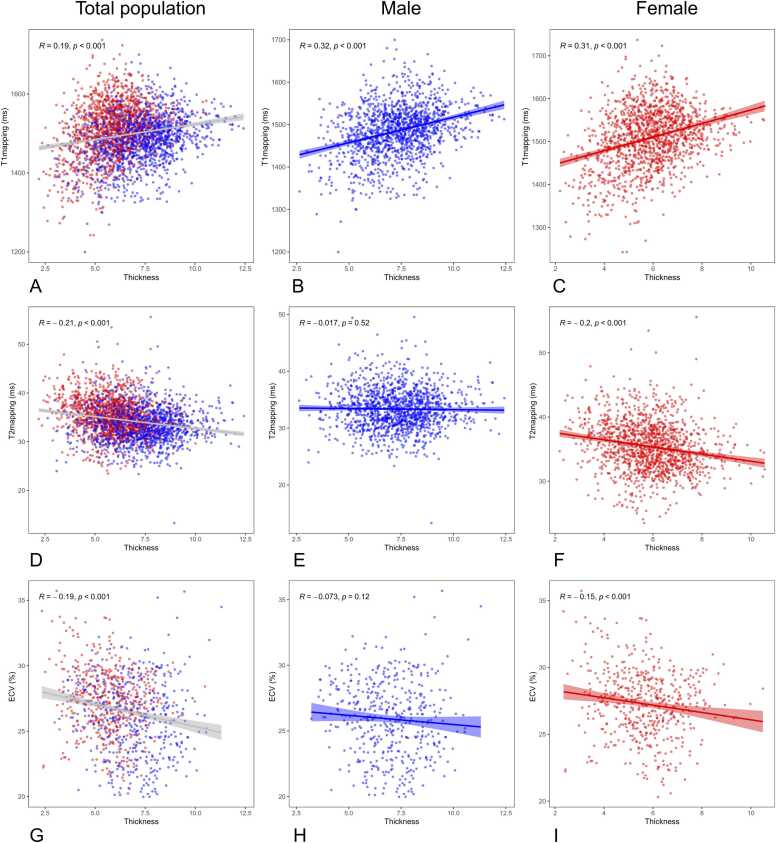
Regression plots of myocardial native T1 values, T2 values, and ECV according to myocardial thickness. Scatterplots show the relationship between myocardial thickness and native T1 values (A-C), T2 values (D-F), and ECV values (G-I) in the total cohort (A, D, G), male participants (B, E, H), and female participants (C, F, I), respectively. *ECV* extracellular volume

The relationships between segmental myocardial T1, T2, and ECV values and myocardial thickness are shown in Fig. 7. Across the total cohort, linear regression analysis demonstrated a positive correlation between T1 values and myocardial thickness (*r* = 0.19, *p* <.001), and negative correlations between T2 values (*r* = −0.21, *p* <.001), ECV (*r* = −0.19, *p* <.001), and myocardial thickness. A significant negative correlation between myocardial thickness and T2 values (*r* = −0.20, *p* <.001) and between myocardial thickness and ECV (*r* = −0.15, *p* <.001) was observed in female participants but not in male participants. In contrast, the positive correlation between T1 values and myocardial thickness remained significant in both male (*r* = 0.32, *p* <.001) and female (*r* = 0.31, *p* <.001) subgroups.

### 3.6 Comparison with 1.5T and 3.0T cardiac MR

Table 5 compares the normal myocardial native T1, T2, and ECV values obtained at 5.0T in this study with previously reported values at 1.5T and 3.0T MR system[15]. The mean native T1 value at 5.0T (1496 ± 37 ms) was markedly higher than those at 3.0T (1170 ± 61 ms, 95% CI: 1164–1176 ms) and 1.5 T (958 ± 43 ms, 95% CI: 957–959), with both *p* <.001. The corresponding Cohen’s d values indicated large positive effect sizes (5.98 for 5.0T vs 3.0T; 12.45 for 5.0T vs 1.5T). In contrast, the mean T2 value at 5.0T (35 ± 3 ms) was significantly lower than those at 3.0T (42 ± 3 ms, 95% CI: 41–42 ms) and 1.5 T (52 ± 3 ms, 95% CI: 51–53 ms) (*p*<.001 for both comparisons), with corresponding large negative effect sizes (−6.76 for 5.0T vs 3.0T; −17.1 for 5.0T vs 1.5T). There were no significantly difference between mean ECV at 5.0T (27 ± 2%) and the reported values at both 3.0T (27 ± 2%, 95% CI: 27–27) and 1.5T (27± 4%, 95% CI: 27–28).

**Table 5:** Comparison of myocardial native T1, T2, and ECV values at 5.0T with previously reported values at 3.0T and 1.5T.

Parameters	Present study values^a^	Reported values^a^		5.0T vs 3.0T		5.0T vs 1.5T	
	5.0T	3.0T	1.5T	*p* value	Cohen's d	*p* value	Cohen's d
T1 mapping (msec)	1496±37	1170±61	958±43	<.001	5.98	<.001	12.49
T2 mapping (msec)	35±3	42±3	52±3	<.001	-2.6	<.001	-6.29
ECV (%)	27±2	27±2	27±4	.812	-0.02	.15	-0.15

Note: *ECV* extracellular volume

a Data are means ± standard deviations

## 4 Discussion

This study was designed to establish reference ranges for myocardial native T1 and T2 relaxation times in healthy volunteers at 5.0T and to investigate their associations with age, sex, heart rate, BMI, and myocardial thickness. Myocardial native T1 and T2 values were significantly higher in female participants compared with male participants. Native T1 values showed a positive correlation with myocardial thickness, whereas native T2 values and ECV demonstrated negative correlations with myocardial thickness. In addition, a significant negative correlation was observed between heart rate and T2 values. Native T1, T2, and ECV values exhibited significant negative correlations with BMI. No significant correlations were observed between native T1, T2, or ECV values and age in the overall cohort.

In vitro phantom experiments demonstrated that T1 and T2 values obtained using the MOLLI sequence and T2-prepared single-shot imaging were comparable to the standard T1 and T2 values obtained using IR-FSE T1 and SEME T2 sequences, indicating good reliability and stability of these techniques at 5.0T. Both quantitative goodness-of-fit analysis and rigorous qualitative assessments demonstrated highly reliable pixel-wise parameter estimation, underscoring the robust and acceptable image quality of myocardial mapping at 5T. A small sample size study by Guo *et al.* [16] further confirmed the feasibility of T1 mapping quantification at 5.0T. It is well established that myocardial T1 measurements are approximately 30%–40% higher and T2 times are approximately 20% lower at 3.0T compared with 1.5T [17]. Consistent with the known effects of field strength on relaxation times, our in vivo results demonstrated that native T1 values at 5.0T were higher and native T2 values were lower than reference values reported for both 1.5T and 3.0T CMR. This pattern aligns with findings from Dabir *et al.* [18], Roy *et al.* [17] and Kawel *et al.* [19]. The 5T ECV values demonstrated no statistically significant differences from the previously reported reference values at 3T and 1.5T. This aligns with the findings of Kawel et al. [20] regarding ECV at 1.5T and 3T. Furthermore, these results indicate the stability of the 5T native T1 mapping and post-T1 mapping sequences.

The clinical utility of mapping techniques critically depends on the reproducibility of quantitative results. Our study demonstrates that myocardial T1, T2, and ECV measurements exhibit excellent intra- and inter-observer reproducibility, indicating that current cardiac mapping techniques of 5.0T MRI meet the requirements for clinical application. Although differences between some segments did not reach statistical significance, evident segmental heterogeneity was still observed as follows: the septum and midventricular segments showed the most consistent agreement, whereas the inferior wall and apical regions displayed relatively higher variability, particularly evident in T1 and T2 mapping. This aligns with the known susceptibility of these regions to motion, partial-volume effects, and magnetic field inhomogeneity [21], [22].

An interesting finding of our study was the relative age-independence of myocardial native T1, T2, and ECV values, except for a significant inverse association between global T1 values and age in female participants. Previous studies have reported inconsistent results regarding age-related changes in myocardial tissue properties. The Multi-Ethnic Study of Atherosclerosis (MESA) [23] and several small series [17], [24] demonstrated an age-dependent increase in myocardial T1 and ECV values in male participants, whereas, Rauhalammi *et al.* [25] reported an inverse association between global T1 values and age in female participants. A large multicenter study found no significant influence of age on either T1 or T2 values [26]. By contrast, Rosmini *et al.* [27] showed that myocardial T1 values measured by both MOLLI and short MOLLI techniques were slightly lower with increasing age, consistent with our findings. And Cadour *et al.* demonstrated that myocardial T1, T2, and T2* values are strongly modulated by sex and age: women exhibit higher T1 and T2 values, particularly during youth, and all three parameters decline with advancing age—findings that align closely with our own observations[28]. This phenomenon may be attributed to the age-related accumulation of myocardial lipofuscin or hemosiderin, which can shorten T1 relaxation times. The inconsistency in research findings could be due to the subtle nature of these changes in healthy volunteers, where the wider normal range of T1 values makes such minor variations more readily observable compared to T2 values. Additionally, the scarcity of large-scale normative data further exacerbates this challenge.

Besides age, gender may be a factor influencing myocardial mapping parameters. Our study found significant sex differences in both myocardial native T1 and T2 values, with female participants exhibiting higher T1 and T2 values than male participants, while no sex-related differences were observed in ECV. These findings are partially consistent with previous studies. Dong *et al.* [29], Roy *et al* [17] and the MESA study [23] reported higher native T1 and ECV values in females compared with males, and Piechnik *et al.* [30] also demonstrated higher native T1 values in females at 1.5T in a cohort of 342 healthy subjects. In contrast, other studies, including those by Dabir *et al.* [18], Liu *et al.* [31], and von Knobelsdorﬀ-Brenkenhoﬀ *et al.* [32], did not find significant sex-related differences in either native T1 or ECV values. Regarding T2 mapping, Bönner *et al.* [33] and Xu *et al.* [26] reported significantly higher T2 values in female participants, consistent with our findings. However, no sex differences in T2 values were observed in studies by von Knobelsdorﬀ-Brenkenhoﬀ *et al.* [32], and Roy *et al.* [17]. Several factors may account for the observed variability across studies. Some prior studies had unequal numbers of male and female participants, potentially biasing sex comparisons. Although earlier hypotheses suggested that greater cardiac motion in females might contribute to increased T2 variability, we found no significant difference in heart rate between sexes in our cohort (65.51 ± 10.30 bpm for male vs 66.15 ± 9.72 bpm for female; *p* =.670). Hormonal differences, particularly androgen effects, may contribute to myocyte hypertrophy, alterations in glycogen content, and changes in capillary density, leading to higher T2 values in females [34]. In addition, males experience a significant reduction in myocardial cell numbers with aging, potentially resulting in increased myocardial fiber size via myocyte fusion and a relative reduction in interstitial space [35], which could explain the higher native T1 values observed in females.

Physiological factors also influence myocardial tissue characteristics. The relationship between heart rate and T2 relaxation time remains a topic of discussion. While several studies have reported no association between heart rate and T2 values in healthy subjects [17], [26], [36], others, in line with our results, have observed lower T2 values in patients with higher heart rates [28], [32]. One possible explanation is that elevated heart rates may exacerbate incomplete T1 recovery effects, which could influence T2 mapping. This observation holds clinical relevance, as subtle T2 elevations—potentially indicative of pathology—might be masked in patients with tachycardia. In our study, significant negative correlations were observed between BMI and myocardial native T1, T2, and ECV values. Similarly, Piechnik *et al.* [30] reported a significant inverse correlation between midwall myocardial T1 values and BMI. However, other studies have reported no significant associations between mapping values and BMI [26], [37], [38]. A possible explanation for the lower myocardial T1 and T2 values observed in individuals with higher BMI is that increased myocardial fat deposition may occur in parallel with greater epicardial fat accumulation [39]. Furthermore, emerging evidence indicates that the mechanisms underlying the association between BMI and myocardial relaxation parameters are multifaceted. In particular, reductions in myocardial water content [28] and alterations in autophagy‑related pathways [40] have been implicated as crucial drivers linking adiposity to myocardial tissue changes. Consequently, the lower myocardial T1 and T2 values observed in individuals with higher BMI likely result from the combined effects of these intricate metabolic and structural adaptations, and this phenomenon may be further accentuated at the high magnetic field strength of 5.0T MR.

Several previous studies have reported regional variations in myocardial mapping parameters across different left ventricular levels. Bönner *et al.* [33] demonstrated that T2 values at the apical short-axis slices were significantly higher than those at the basal slices in both male and female volunteers. Similarly, Dong *et al.* [29] and von Knobelsdorﬀ-Brenkenhoﬀ *et al.* [32] reported that myocardial native T1 and ECV values increased progressively from the base to the apex. These observations have been attributed to the partial volume effect arising from the curvature of the left ventricle, resulting in inclusion of blood pool signal within myocardial voxels [32]. In contrast, our study observed slightly higher T2 values and ECV values, but lower T1 values, at the apex compared with the basal and midventricular myocardium. Given that partial volume effects would be expected to elevate both T1 and T2 values simultaneously, the pattern observed in our study cannot be solely explained by this phenomenon. Instead, we found that regional variations in T1, T2, and ECV values were significantly correlated with segmental myocardial thickness, suggesting that intrinsic myocardial tissue characteristics may contribute to these differences. Factors such as myocardial thickness, collagen content, fiber orientation, and regional perfusion may influence segmental mapping values under normal conditions. Although the observed differences across segments were relatively minor, they must be carefully considered in clinical applications, particularly given the potential for overlap between normal and pathological tissue. This consideration is critical when evaluating cardiomyopathies that typically demonstrate regional involvement, such as apical HCM, myocarditis, and Fabry disease.

## 5 Limitations

This study has several limitations. First, this single-center study exclusively used a 5.0T United Imaging scanner in Chinese adults, lacking multi-ethnic, multi-site, and multi-vendor validation, which may limit the generalizability of the reference ranges. Second, this study was based on well-screened asymptomatic volunteers, but potential selection bias cannot be entirely excluded; furthermore, despite comprehensive clinical screening and transthoracic echocardiographic assessment, the possibility of subclinical disease could not be fully ruled out. Third, contrast-enhanced imaging was not performed in all participants, potentially introducing bias in ECV assessment. Fourth, incidental myocardial fibrosis undetectable by echocardiography may have influenced the results. Finally, in the absence of relevant reports on 5.0T CMR mapping, more cases were not included to demonstrate its diagnostic ability, which will be addressed in our future study. During enrollment, three patients with ICM, HCM, and myocardial amyloidosis were found by accident. We also performed CMR examination of these patients, and their mapping and LGE images can effectively display lesions.

## 6 Conclusion

In summary, we established reference ranges for myocardial native T1, T2 relaxation times, and ECV in healthy Chinese adults at 5.0T for the first time. These values were significantly different from those previously reported at 3.0T and 1.5T. Myocardial native T1 and T2 values were found to be dependent on sex, BMI, and myocardial thickness, and segmental variations in myocardial mapping values were also observed.

## Funding

Joint Research Development Project between Shenkang and United Imaging on Clinical Research and Translation (SKLY2022CRT201); The Shanghai Municipal “Explorer Plan” (the second batch) project in 2024 (24TS1411000); Natural Science Foundation of Fujian Province (2022J05333); Shanghai Pujiang Program (21PJD012).

## Author contributions

**Shiyu Wang:** Writing – review & editing, Writing – original draft, Visualization, Validation, Supervision, Software, Resources, Project administration, Methodology, Investigation, Formal analysis, Data curation, Conceptualization. **Xianling Qian:** Writing – review & editing, Writing – original draft, Visualization, Validation, Software, Resources, Project administration, Methodology, Investigation, Formal analysis, Conceptualization. **Yali Wu:** Writing – original draft, Visualization, Software, Methodology, Investigation, Formal analysis, Conceptualization. **Xiyin Miao:** Resources, Project administration, Methodology, Data curation. **Ziyun Guan:** Software, Resources, Project administration, Methodology. **Dong Wang:** Validation, Software, Project administration, Methodology. **Rui Wang:** Visualization, Resources, Methodology. **Yinyin Chen:** Supervision, Project administration, Funding acquisition. **Ling Chen:** Investigation, Data curation. **Zhuolin Liu:** Investigation, Formal analysis. **Shaofeng Duan:** Visualization, Validation. **Lin Tian:** Software, Methodology. **Hang Jin:** Writing – review & editing, Visualization, Validation, Supervision, Software, Resources, Project administration, Funding acquisition, Conceptualization. **Mengsu Zeng:** Writing – review & editing, Visualization, Validation, Supervision, Software, Resources, Project administration, Methodology, Investigation, Funding acquisition, Formal analysis, Data curation, Conceptualization.

## Disclosure

Authors Z.Y.G, D.W., R.W., and S.F.D are employees of United Imaging, the manufacturers of the 5.0T MRI equipment used in this study, and have provided advice on sequence parameter adjustments. L.T is an employee of Circle Cardiovascular Imaging, the manufacturers of cardiac MR analysis software, and have provided technical support for cardiac MR imaging analysis.

## Data Availability

The dataset generated in this study was designed to serve as a normative reference for myocardial native T1, T2, and extracellular volume measurements at 5.0T. Due to institutional ethics regulations and data protection policies, individual-level imaging data are not publicly available. Anonymized data supporting the findings of this study are available from the corresponding author upon reasonable request and subject to institutional approval.

## Footnotes

Appendix A Supplementary data associated with this article can be found in the online version at doi:10.1016/j.jocmr.2026.102753.
