# Supplementary material for: Reference ranges for myocardial native T1, T2, and extracellular volume at 5.0T cardiac magnetic resonance imaging in healthy adults
Source: J Cardiovasc Magn Reson. 2026 Jun 4;28(2):102753. doi: 10.1016/j.jocmr.2026.102753 (PMC13292681; doi:10.1016/j.jocmr.2026.102753)
Supplement: Supplementary file 1 — Supplementary material [file mmc1.docx]

**Supplemental Materials**

**Table S1:** 5.0T MRI acquisition parameters of phantom experiments

| Parameters | IR-FSE T1 | MOLLI T1map | SEME T2 | T2 prep |
| --- | --- | --- | --- | --- |
| Thickness (mm) | 3 | 8 | 5 | 8 |
| Repetition time (msec) | 10000 | 4.58 | 2348 | 3.851 |
| Echo time (msec) | 9.62 | 1.72 | 12.5,25,37.5,50,62.5,75, 87.5,100 | 1.425 |
| T2prep (msec) | NA | NA | NA | 0,100,200 |
| Inversion time (msec) | 75,100,125,150,200,300,500,800,1000, 1500,2000,2500 | 155, 155+RR, 155+2RR,155+3RR,155+4RR  235,235+RR,235+2RR | NA | NA |
| Flip angle (º) | 130 | 7 | 150 | 5 |
| Matrix | 256×320 | 128×128 | 250×288 | 108×144 |
| Bandwidth (Hz) | 260 | 400 | 250 | 600 |
| Field of view (mm) | 250×250 | 200×200 | 200×230 | 200×200 |
| Pixel size (mm) | 0.78×0.78 | 1.56×1.56 | 0.8×0.8 | 1.85×1.39 |
| Total time (s) | 4320 | 11.3 | 286 | 10.8 |

Note: MRI = magnetic resonance imaging, IR-FSE T1 = Inversion Recovery Fast Spin Echo T1-weighted Imaging, MOLLI = Modified Look-Locker Inversion recovery, SEME T2 = Spin Echo Multi-Echo T2-weighted Imaging, NA = not applicable. RR = R‑R interval; virtual ECG is used in phantom experiments with RR interval = 800 ms.

**Table S2:** 5.0T CMR T1 mapping and T2 mapping sequence parameters

| Parameters | Pre-contrast T1mapping | Post-contrast T1 mapping | T2 mapping |
| --- | --- | --- | --- |
| Method/Pattern | MOLLI/5(3)3 | MOLLI/4(1)3(1)2 | T2 Preparation based/1(3)1(3)1 |
| Thickness (mm) | 8 | 8 | 8 |
| Repetition time (msec) | 3.99 | 3.99 | 4.22 |
| acquisition window(msec) | 295 | 295 | 342 |
| Echo time (msec) | 1.45 | 1.45 | 1.50 |
| Inversion time (msec) | 155, 155+RR, 155+2RR,155+3RR,155+4RR,235,235+RR,235+2RR | 155, 155+RR, 155+2RR, 155+3RR,235,235+RR,235+2RR,315,315+RR | NA |
| T2prep (msec) | NA | NA | 0,30,50 |
| Flip angle (°) | 7 | 7 | 9 |
| Matrix | 228×256 | 228×256 | 185×208 |
| Bandwidth (Hz) | 800 | 800 | 600 |
| Field of view (mm) | 320×360 | 320×360 | 320×360 |
| Pixel size (mm) | 1.40×1.40 | 1.40×1.40 | 1.73×1.73 |
| Imaging acceleration | PI 2 | PI 2 | PI 2 |
| Heart beats per slice | 11 | 11 | 9 |

Note: CMR= cardiac magnetic resonance, NA = not applicable, MOLLI = Modified Look-Locker Inversion recovery,

RR = R‑R interval，PI = Parallel imaging.

**Table S3:** T1 values measured by IR-FSE and MOLLI T1 mapping in phantom study

| Sample number | IR-FSE T1 (msec) | MOLLI T1map (msec) |
| --- | --- | --- |
| 1 | 331.1 | 438.2 |
| 2 | 491.8 | 492 |
| 3 | 706.8 | 742.7 |
| 4 | 942.4 | 945.8 |
| 5 | 1200 | 1179.7 |
| 6 | 1385.3 | 1322.3 |
| 7 | 1815.6 | 1748 |
| 8 | 2261.8 | 2146.2 |
| 9 | 2615.3 | 2501.8 |
| 10 | 2850.4 | 2870.6 |

Note: IR-FSE T1 = Inversion Recovery Fast Spin Echo T1-weighted Imaging, MOLLI = Modified Look-Locker Inversion recovery.

**Table S4:** T2 values measured by SEME T2 and T2-prepared single-shot imaging in phantom study

| Sample number | SEME T2 (msec) | T2 prep (msec) |
| --- | --- | --- |
| 1 | 17 | 16.8 |
| 2 | 26.7 | 28.7 |
| 3 | 39.1 | 36.3 |
| 4 | 55.9 | 61 |
| 5 | 76.9 | 84.3 |
| 6 | 94.7 | 93 |
| 7 | 150.6 | 150 |

Note: SEME T2 = Spin Echo Multi-Echo T2-weighted Imaging.

**Table S5:** Intra- observer and Inter-observer agreement of native T1-mapping, T2-mapping, and post T1-mapping measurements

| Sequence | ICC | |
| --- | --- | --- |
|  | Intra- observer | Inter-observer |
| Native T1-mapping | 0.996 | 0.997 |
| T2-mapping | 0.993 | 0.994 |
| ECV mapping | 0.989 | 0.990 |

Note: ICC = intraclass correlation coefficient.

**Table S6:** Inter-observer Agreement of segmental T1, T2 and ECV Measurements

| AHA 16- segment model | ICC | | |
| --- | --- | --- | --- |
|  | T1 | T2 | ECV |
| 1 | 0.959 | 0.917 | 0.973 |
| 2 | 0.934 | 0.960 | 0.991 |
| 3 | 0.985 | 0.982 | 0.973 |
| 4 | 0.952 | 0.964 | 0.973 |
| 5 | 0.968 | 0.975 | 0.957 |
| 6 | 0.972 | 0.969 | 0.968 |
| 7 | 0.979 | 0.979 | 0.953 |
| 8 | 0.994 | 0.990 | 0.970 |
| 9 | 0.993 | 0.987 | 0.965 |
| 10 | 0.964 | 0.952 | 0.977 |
| 11 | 0.948 | 0.972 | 0.914 |
| 12 | 0.976 | 0.983 | 0.947 |
| 13 | 0.888 | 0.960 | 0.975 |
| 14 | 0.969 | 0.900 | 0.969 |
| 15 | 0.877 | 0.932 | 0.951 |
| 16 | 0.908 | 0.966 | 0.982 |

Note: AHA = American Heart Association, ICC = intraclass correlation coefficient

**Table S7:** Subjective overall image quality assessment across native T1-, T2-, and ECV mapping sequences

| AHA 16- segment model | Native T1-mapping | T2-mapping | ECV-mapping |
| --- | --- | --- | --- |
| 1 | 4.71 ± 0.50 | 4.90 ± 0.31 | 4.92 ± 0.33 |
| 2 | 4.70 ± 0.49 | 4.76 ± 0.44 | 4.89 ± 0.37 |
| 3 | 4.74 ± 0.47 | 4.75 ± 0.46 | 4.89 ± 0.37 |
| 4 | 4.63 ± 0.52 | 4.69 ± 0.51 | 4.85 ± 0.44 |
| 5 | 4.64 ± 0.53 | 4.68 ± 0.49 | 4.87 ± 0.43 |
| 6 | 4.70 ± 0.49 | 4.85 ± 0.37 | 4.92 ± 0.33 |
| 7 | 4.93 ± 0.28 | 4.92 ± 0.30 | 4.92 ± 0.33 |
| 8 | 4.90 ± 0.36 | 4.81 ± 0.44 | 4.92 ± 0.33 |
| 9 | 4.92 ± 0.33 | 4.84 ± 0.41 | 4.92 ± 0.33 |
| 10 | 4.78 ± 0.44 | 4.76 ± 0.44 | 4.87 ± 0.43 |
| 11 | 4.78 ± 0.44 | 4.75 ± 0.48 | 4.85 ± 0.44 |
| 12 | 4.93 ± 0.28 | 4.90 ± 0.31 | 4.93 ± 0.31 |
| 13 | 4.97 ± 0.20 | 4.89 ± 0.35 | 4.85 ± 0.40 |
| 14 | 4.97 ± 0.21 | 4.73 ± 0.47 | 4.85 ± 0.40 |
| 15 | 4.97 ± 0.24 | 4.78 ± 0.47 | 4.85 ± 0.40 |
| 16 | 4.97 ± 0.24 | 4.79 ± 0.46 | 4.89 ± 0.37 |
| Global | 4.49 ± 0.58 | 4.36 ± 0.58 | 4.74 ± 0.51 |

**Note:** AHA = American Heart Association.

**Table S8:** Goodness-of-fit (R^2^) values for T1 mapping across different slices

|  | Myocardium | Blood pool |
| --- | --- | --- |
| basal | 0.998[0.996,0.998] | 0.998[0.997,0.998] |
| mid | 0.998[0.997,0.998] | 0.998[0.997,0.998] |
| apical | 0.998[0.997,0.999] | 0.998[0.997,0.999] |


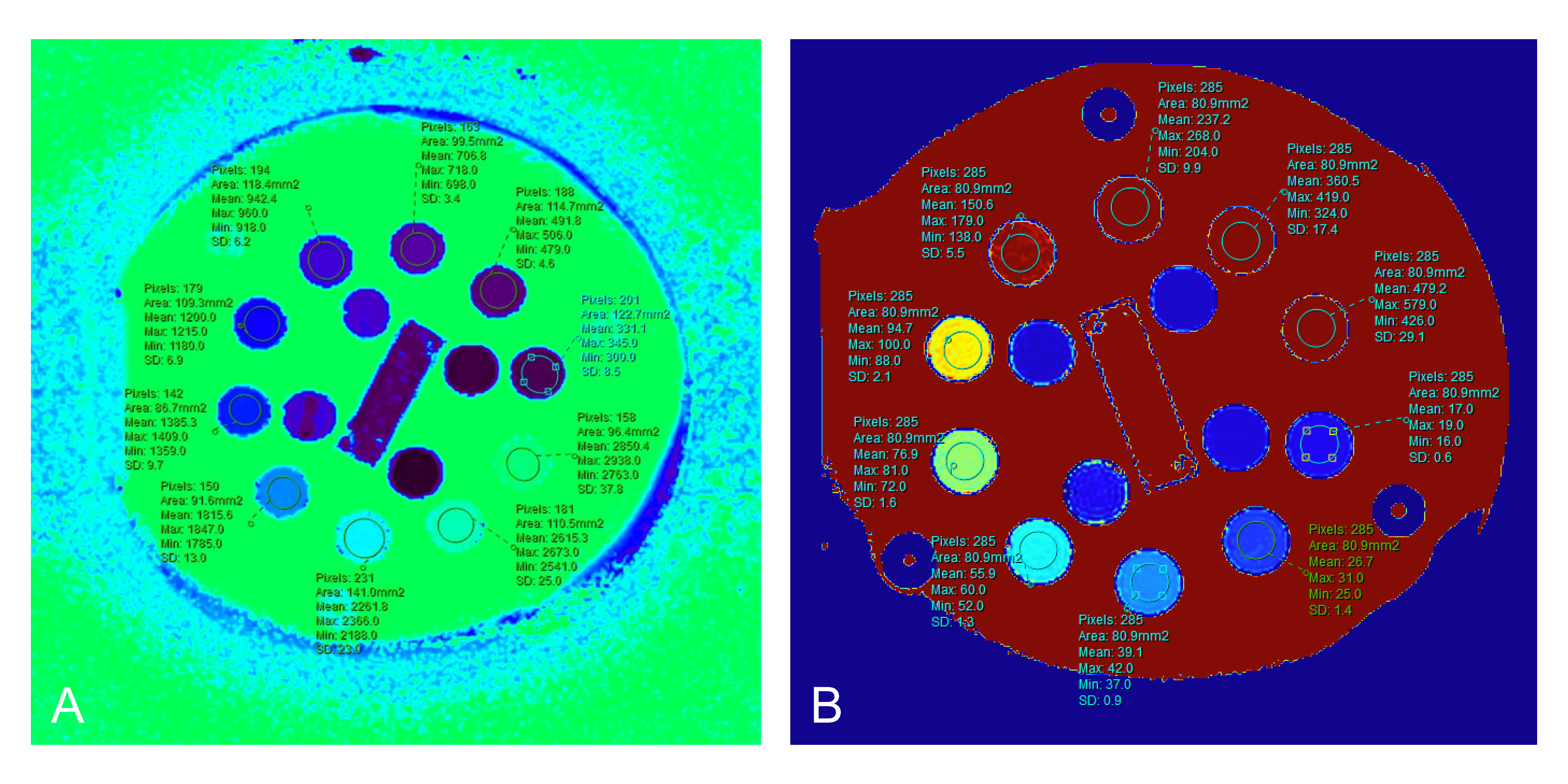


**Figure S1:** Phantom T1 and T2 mapping at 5.0T MR. (**A**) T1 values of each phantom sphere measured using inversion recovery fast spin-echo (IR-FSE) T1-weighted imaging. (**B**) T2 values of each phantom sphere measured using spin-echo multi-echo (SEME) T2-weighted imaging.


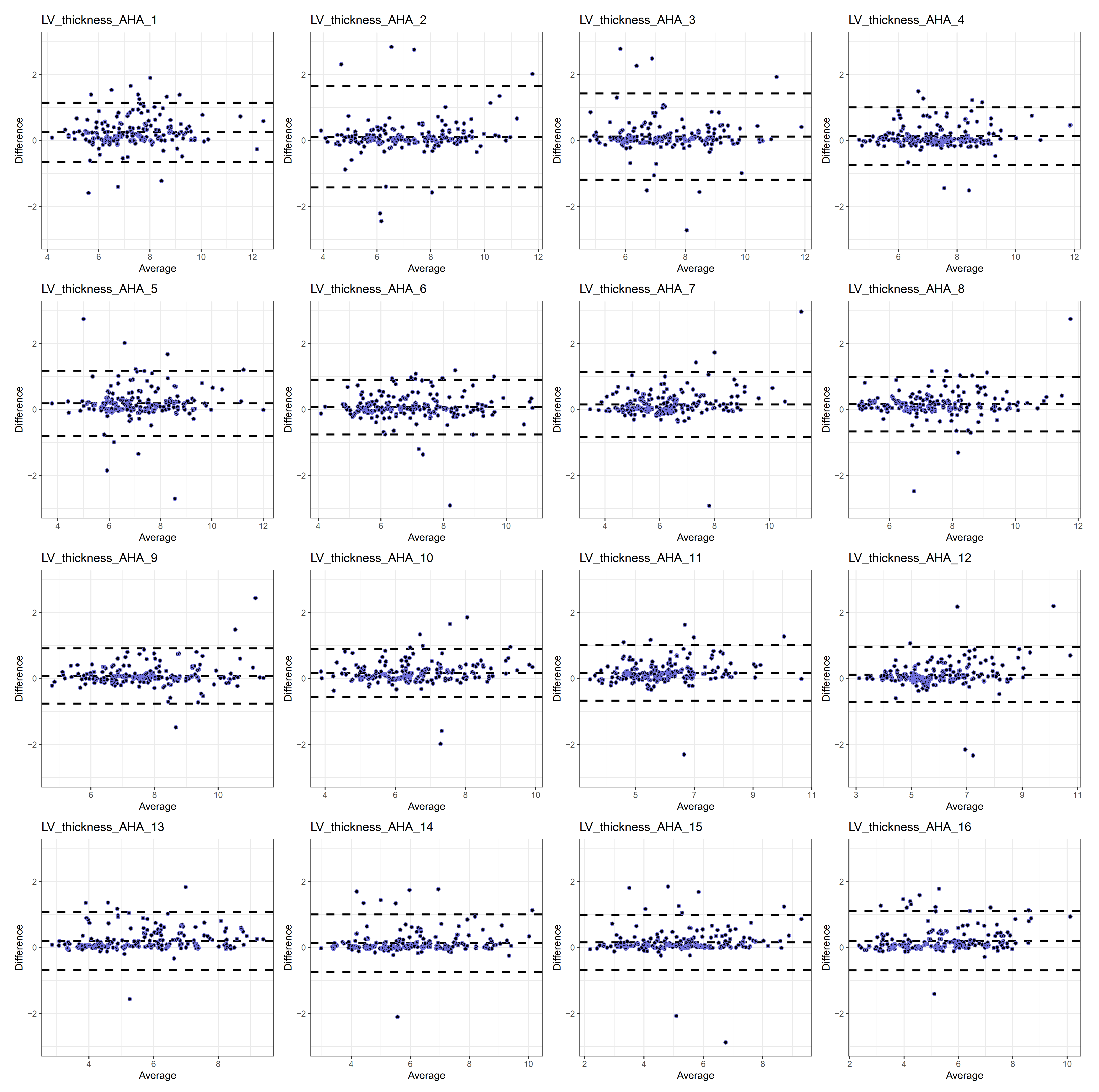


**Figure S2:** Bland-Altman plots illustrating interobserver agreement for segmental myocardial thickness measurements based on the AHA 16-segment model.


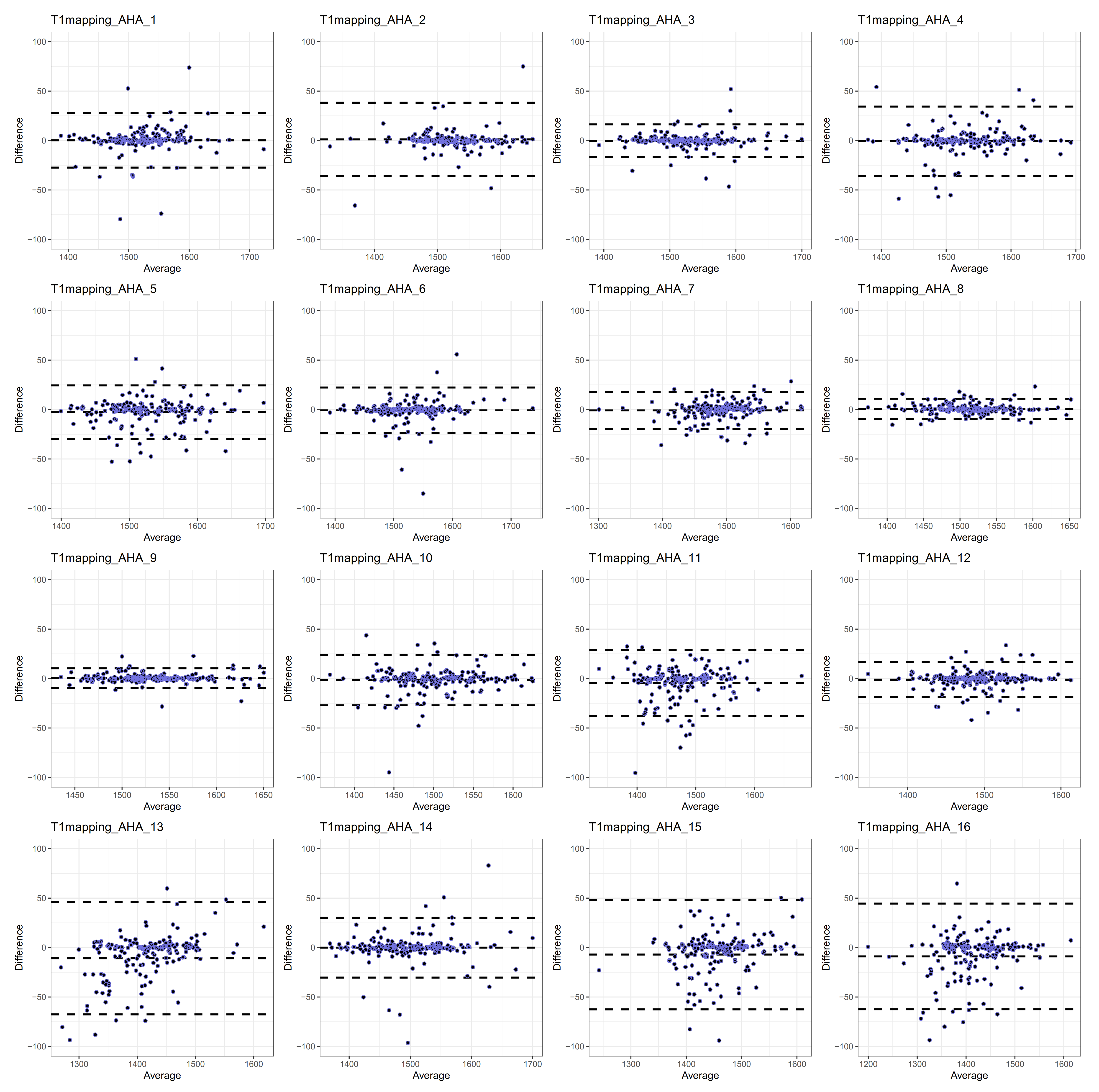


**Figure S3:** Bland-Altman plots illustrating interobserver agreement for segmental myocardial T1 value measurements based on the AHA 16-segment model.


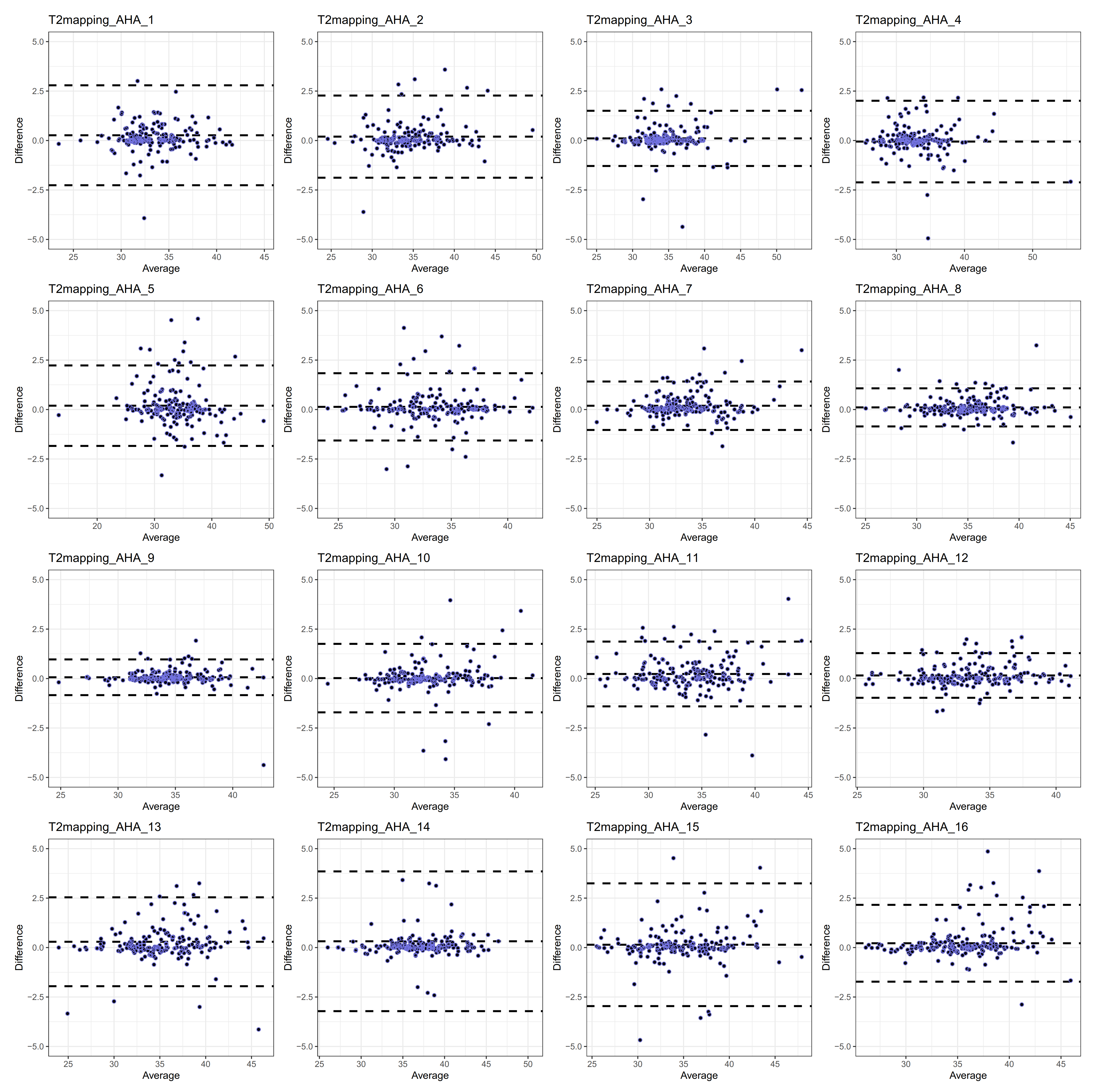


**Figure S4:** Bland-Altman plots illustrating interobserver agreement for segmental myocardial T2 value measurements based on the AHA 16-segment model.


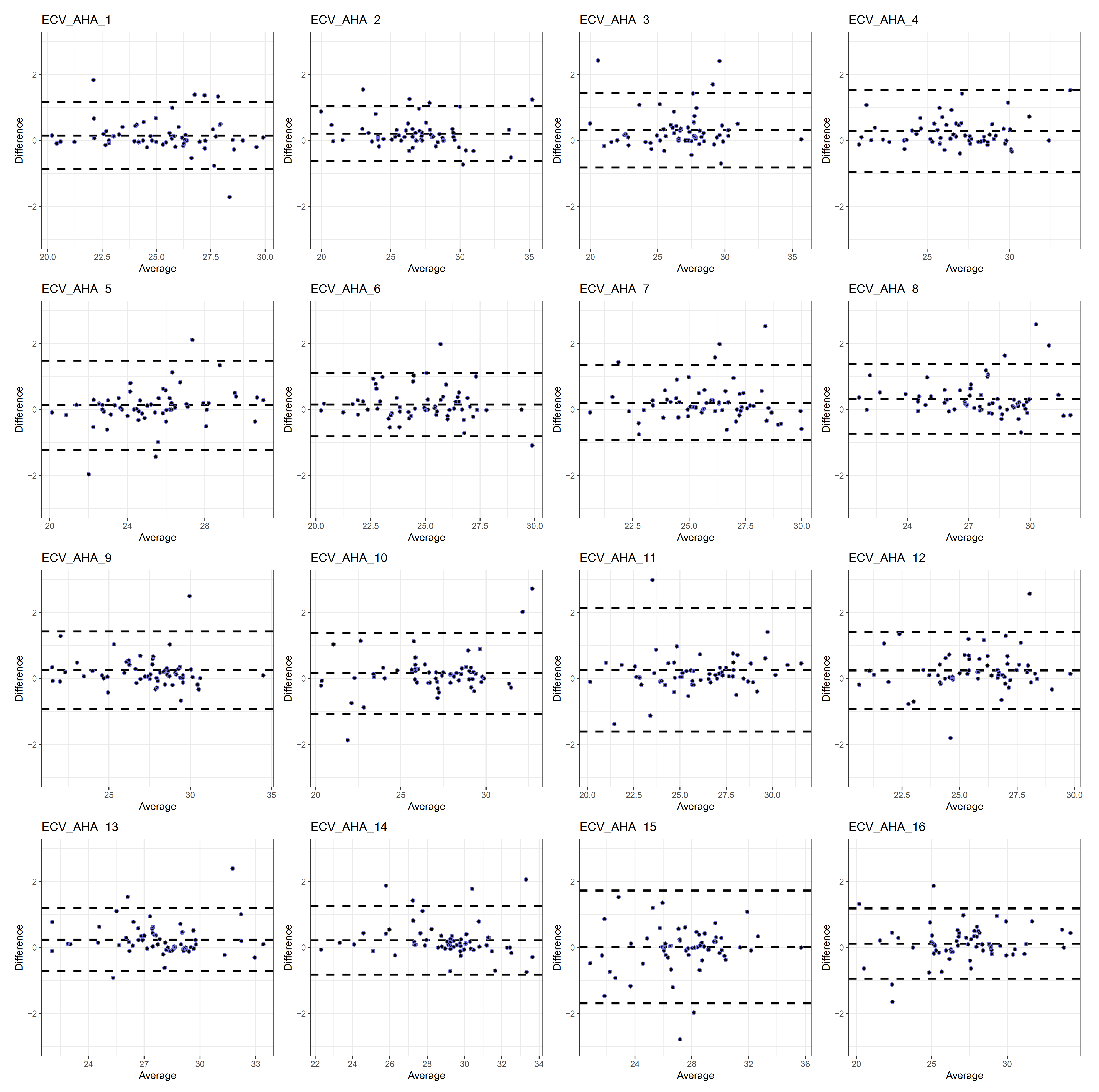


**Figure S5:** Bland-Altman plots illustrating interobserver agreement for segmental myocardial extracellular volume (ECV) measurements based on the AHA 16-segment model.


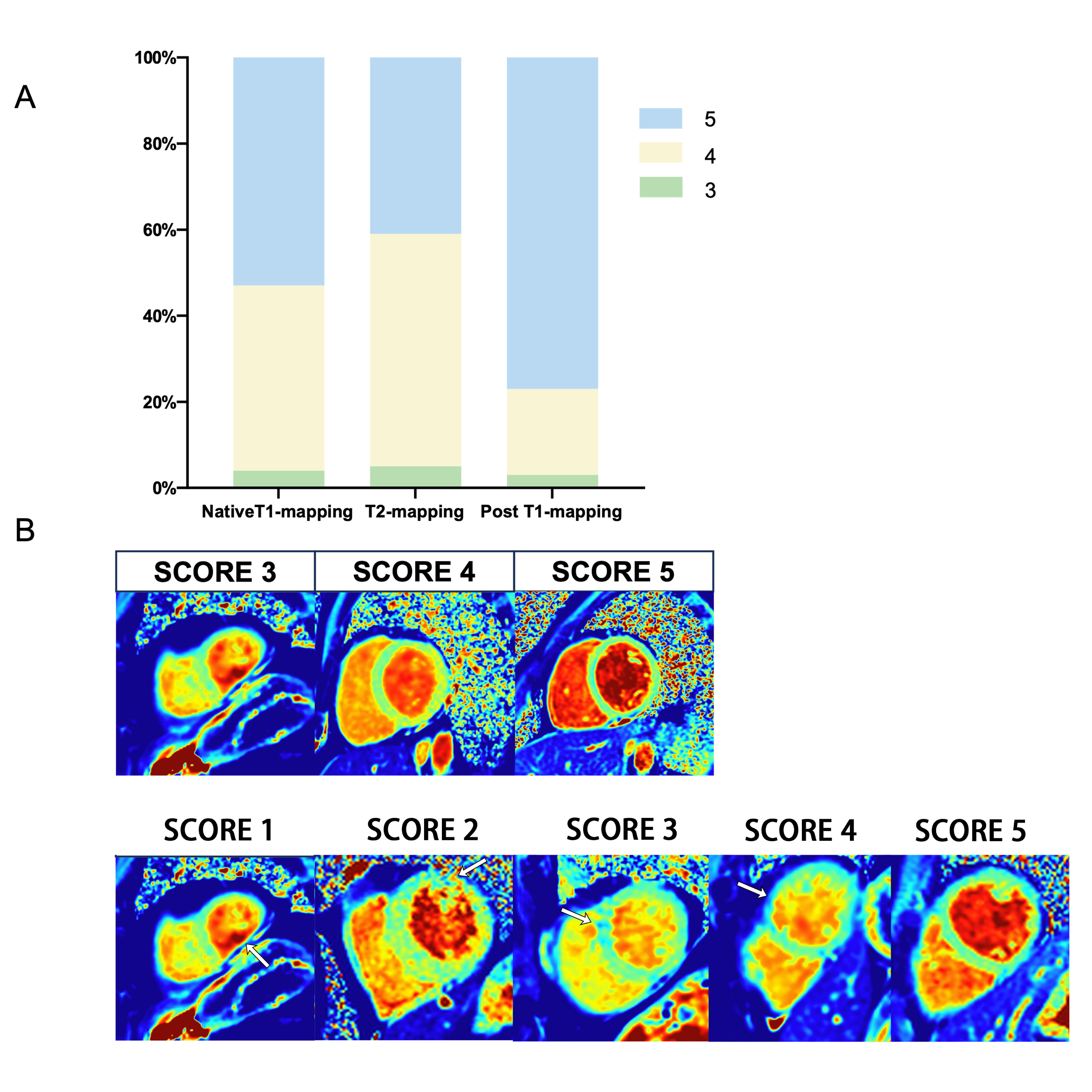


**Figure S6: Distribution of overall image quality scores for mapping sequences (A) and representative examples of scoring criteria (B).** (A) Distribution of overall image quality scores using a five-point Likert scale for native T1-mapping, post T1-mapping, and T2-mapping sequences. (B) Representative short-axis myocardial maps illustrating the quality scoring. The upper row displays examples of overall slice image quality corresponding to Score 3 (good quality), Score 4 (very good quality), and Score 5 (excellent quality). The lower row demonstrates segmental image quality scoring, with white arrows indicating specific myocardial segments evaluated for localized quality. Images or segments with lower scores show less homogeneous signal and increased noise, whereas higher scores reflect sharper borders and improved image quality.


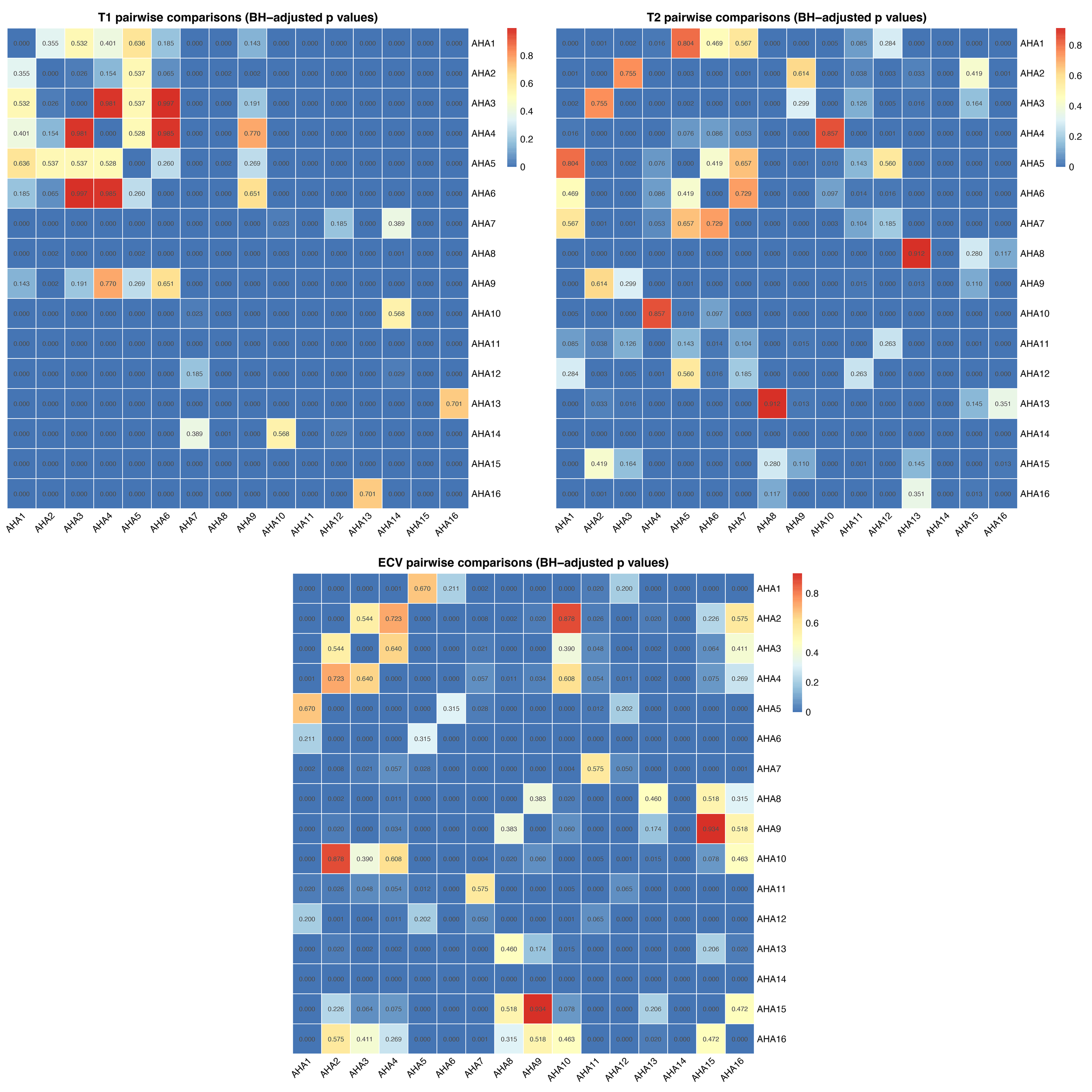


**Figure S7: Pairwise Comparisons of Quantitative T1, T2, and ECV Mapping Values Across 16 AHA Myocardial Segments.** Statistical differences in T1, T2, and ECV values across myocardial segments. Heatmaps illustrate the Benjamini-Hochberg (BH) adjusted p-values from pairwise comparisons of T1 (top left), T2 (top right), and extracellular volume (ECV, bottom) across the 16 American Heart Association (AHA) myocardial segments. The color gradient and numerical values reflect the magnitude of the adjusted p-values, with blue indicating lower p-values (statistically significant difference) and red/yellow indicating higher p-values (no statistically significant difference). This provides a robust statistical framework for evaluating and interpreting regional heterogeneity.

Supplementary Text S1: **Exploratory Analysis of Synthetic Hematocrit and Synthetic Extracellular Volume (ECV)Methodology**

Following the linear regression relationships described in previous literature [1], we explored the feasibility of calculating synthetic hematocrit (HCT) derived from native T1 values of the blood pool, and subsequently computed the synthetic extracellular volume (ECV).

Blood Pool T1 Measurement: Native T1 values of the blood pool were sampled in the left ventricular cavity at the mid-ventricular short-axis slice. A circular region of interest (ROI) with an area of approximately 100 mm² was drawn in the center of the blood pool. Care was taken to strictly avoid papillary muscles, trabeculae, and the blood-myocardium interface to minimize partial volume effects.

Statistical Analysis: We divided the existing 60 enhanced volunteers with available laboratory hematocrit (HCT) data into two groups. The derivation cohort (45 volunteers) was used to establish the linear regression equation between blood pool T1 and laboratory-measured HCT, and to compare their agreement. The validation cohort (15 volunteers) was used to calculate synthetic ECV values and to compare the agreement between synthetic ECV and conventional ECV.

Findings: Within the derivation cohort, our data confirmed a negative correlation between laboratory HCT and blood pool T1 values (r = –0.500, p < 0.001). The resulting linear regression equation was: HCTsyn = 67.28 – 0.012 × T1blood (Supplementary **Figure S7)**. The overall agreement between laboratory-measured hematocrit and synthetic hematocrit was poor (Intraclass Correlation Coefficient [ICC] = 0.35), although the Bland-Altman plot (Supplementary **Figure S8**) indicated relatively good agreement within certain ranges. Meanwhile, in the validation cohort, the agreement between synthetic ECV and conventional ECV was moderate (ICC = 0.73, Supplementary **Figure S9**).


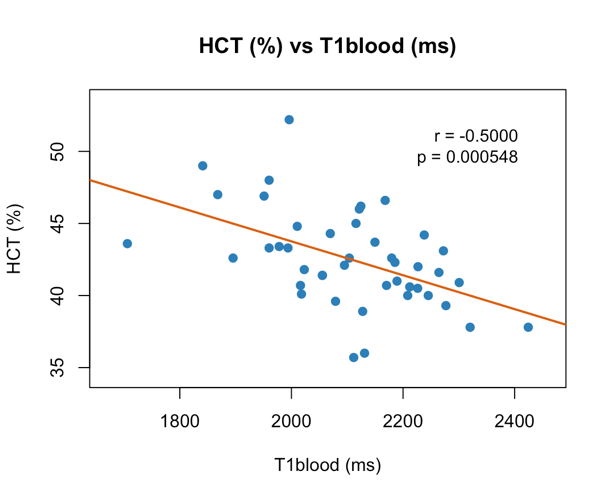


**Figure S7**. Correlation between Hematocrit and Blood Pool T1 Values.


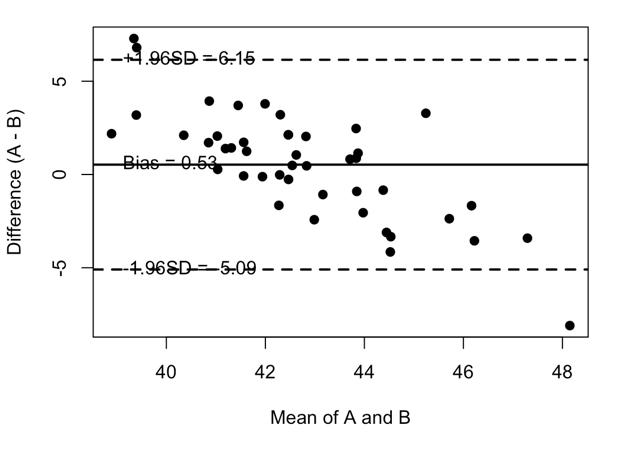


**Figure S8**. Bland-Altman Plot between Synthetic Hematocrit and Laboratory Hematocrit.


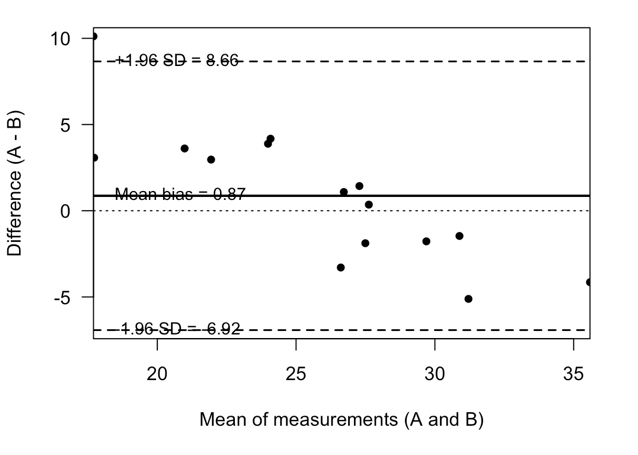


**Figure S9**. Bland-Altman Plot between Synthetic extracellular volume (ECV) and Conventional ECV.

References for Supplementary Material:

Treibel TA, Fontana M, Maestrini V, et al. Automatic Measurement of the Myocardial Interstitium: Synthetic Extracellular Volume Quantification Without Hematocrit Sampling. JACC Cardiovasc Imaging. 2016 Jan;9(1):54-63. doi: 10.1016/j.jcmg.2015.11.008.
